# Complete Blood-Count-Derived Inflammatory Markers Across Acute Mood Episodes

**DOI:** 10.31083/AP45450

**Published:** 2026-04-24

**Authors:** Pelin Göksel, Selçuk Özdin

**Affiliations:** ^1^Department of Psychiatry, Ondokuz Mayıs University, 55270 Samsun, Turkey

**Keywords:** bipolar disorder, depression, BD and MD inflammation, mood disorders

## Abstract

**Background:**

Recent evidence has suggested that low-grade systemic inflammation may contribute to the clinical expression of mood disorders, yet findings have differed considerably across studies. The present study compared several complete blood-count (CBC)-derived inflammatory indices among inpatients with bipolar disorder (BD), manic and depressive episodes, and major depressive disorder (MDD), and examined whether psychotropic medications influenced these inflammatory markers.

**Methods:**

This retrospective chart-review study included only inpatients hospitalized with a diagnosis of BD or MDD between January 1, 2019, and January 1, 2023. A healthy control (HC) group, matched by age and sex, was included for comparison. For data independence, only the first CBC obtained within 24 hours of admission was analyzed. Inflammatory markers, including neutrophil-to-lymphocyte ratio (NLR), monocyte-to-lymphocyte ratio (MLR), platelet-to-lymphocyte ratio (PLR), and systemic immune-inflammation index (SII), were calculated from standardized fasting blood samples. Statistical analyses were performed using the Kruskal-Wallis test for group comparisons, followed by Bonferroni-corrected Mann-Whitney U tests. Additionally, analysis of covariance (ANCOVA) and regression models were employed to control for potential confounders such as age, sex, length of stay, and various psychotropic medications (lithium, antidepressants, and antipsychotics).

**Results:**

NLR and MLR ratios were consistently higher in all mood-disorder groups than in healthy individuals. PLR elevation appeared specific to the MDD group, and the SII was increased in depressive episodes but not in mania. These patterns remained statistically significant after adjustment for demographic covariates; male sex was negatively associated with PLR. Regression analyses adjusted for age and sex demonstrated a negative association between antidepressant use and PLR and MLR values.

**Conclusions:**

These results suggested that acute mood episodes are accompanied by measurable increases in certain inflammation-related hematologic markers. The inverse relationship between antidepressant use and PLR and MLR was consistent with an association between medication use and nonspecific systemic inflammatory markers observed in acutely hospitalized patients.

## Main Points

∙ Neutrophil-to-lymphocyte ratio (NLR) and monocyte-to-lymphocyte ratio (MLR) values were found to be higher in major depressive disorder (MDD), bipolar disorder (BD) manic episode, and BD depressive episode periods than in healthy controls.

∙ Bonferroni-corrected post-hoc comparisons showed no significant differences in NLR or MLR values among the MDD, BD-manic, and BD-depression groups (all *p* > 0.0083).

∙ Antidepressant use was negatively associated with platelet-to-lymphocyte ratio (PLR) and MLR values in regression analyses, no significant relationship was found between the use of other drug groups and the parameters.

## 1. Introduction

Research into the etiology of bipolar disorder (BD) and major depressive disorder (MDD) has suggested a complex interplay of genetic, neuroendocrine, immunological, and psychosocial factors. Although these two distinct psychiatric conditions may share potential biological pathways, uncertainties in their pathophysiology persist [1, 2].

In recent years, low-grade systemic inflammation has been increasingly recognized as a significant factor in the pathogenesis and progression of both BD and MDD [3, 4]. This chronic inflammatory response, characterized by increased pro-inflammatory cytokines such as tumor necrosis factor-α (TNF-α), interleukin-6 (IL-6), and decreased anti-inflammatory cytokines, is thought to trigger a neuroinflammatory process, contributing to the manifestation of psychiatric symptoms [5, 6]. Neuroinflammation has been proposed to be associated with microglial dysfunction in mood disorders, affecting disease severity and treatment response [7, 8].

To evaluate these inflammatory processes, cost-effective markers derived from complete blood-count (CBC) have gained prominence, supplementing traditional cytokine and C-reactive protein (CRP) measurements [9]. The neutrophil-to-lymphocyte ratio (NLR) and the platelet-to-lymphocyte ratio (PLR) are widely used as reliable indicators of systemic inflammation and immune activation [10]. Newer derived markers, such as the systemic immune-inflammation index (SII, calculated as the ratio of platelet count × neutrophil count/lymphocyte count) and the monocyte-to-lymphocyte ratio (MLR), which offer a more complex and comprehensive assessment, have also become a focus of research [11, 12]. High values of such markers support the neuroinflammation hypothesis, indicating pathological immunological processes not only in mood disorders but also in other psychiatric conditions like schizophrenia, sleep disorders, and attention deficit and hyperactivity disorder [13, 14, 15, 16].

The role of inflammatory mechanisms in the etiology of psychiatric disorders is increasingly recognized, and relevant research has suggested that NLR, PLR, and MLR measurements may be markers of neuroinflammatory processes in mood disorders. Although numerous studies have investigated these markers in mood disorders, they have focused on a single-episode type. The lack of a comprehensive comparative analysis that examined patients with BD manic episodes, BD depressive episodes, and MDD within the same framework is a significant shortcoming. Our study, which compared NLR, MLR, PLR, and SII levels in patients hospitalized for BD-manic episode, BD-depression episode, and MDD, both within patient groups and with healthy controls, has the potential to offer important results for both immunology and clinical psychiatry by demonstrating how immunological mechanisms are combined with the clinical manifestations of psychiatric disorders.

## 2. Materials and Methods

**Fig. 1.**
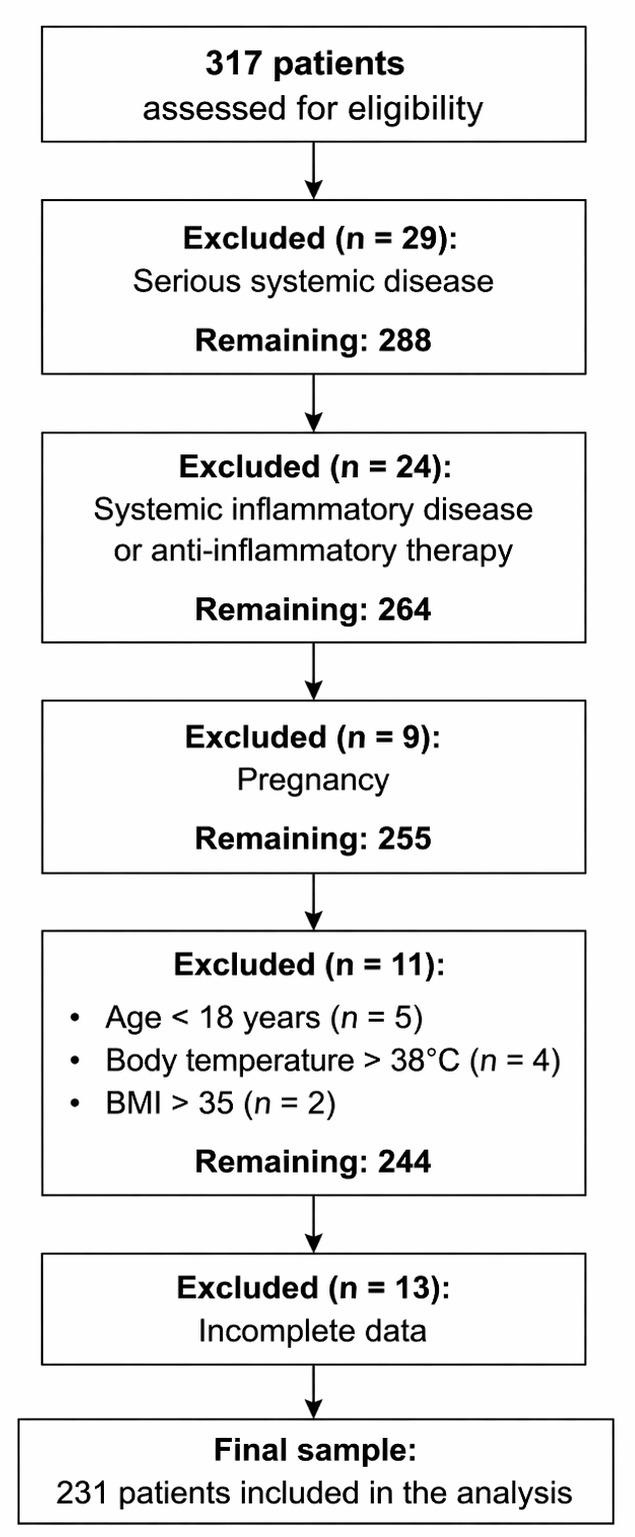
**Participant flow diagram**.

This retrospective study reviewed the records of all inpatients diagnosed with BD-manic episode, BD-depression episode, and MDD at Ondokuz Mayıs Psychiatry Clinic between January 1, 2019 and January 1, 2023. These records included sociodemographic characteristics such as age and sex, DSM-5 (Diagnostic and Statistical Manual of Mental Disorders, 5th Ed.) diagnosis criteria at admission, treatments applied during hospitalization, services provided, comorbidities, and laboratory results. Inclusion criterion: patients over 18 years old who met DSM-5 criteria for BD or MDD. Exclusion criteria: patients with systemic inflammatory disease (e.g., hematologic diseases, chronic obstructive pulmonary disease, ulcerative colitis, autoimmune diseases, rheumatic diseases); those on anti-inflammatory treatments (e.g., corticosteroids, nonsteroidal anti-inflammatory drugs); those with conditions that could cause abnormal parameters (e.g., alcohol/substance use disorder, pregnancy); those with severe systemic diseases (e.g., diabetes, hypertension, liver failure, renal failure); those with a Body Mass Index (BMI) over 35; and those whose body temperature was above 37.5 °C during blood collection. A total of 86 patients were excluded from the study. The participant flow diagram is given in Fig. 1.

The healthy control (HC) group was composed of healthy donors from the same hospital’s blood bank, matched by age and sex to the MDD patient group. Donors with chronic diseases or on medications affecting blood parameters (e.g., nonsteroidal anti-inflammatory drugs, steroids) were excluded.

Because the study had a retrospective design and some patients had multiple admissions or more than one CBC measurement, only independent observations were included in the final analysis. To avoid multiple observations per patient, we applied an index time-point selection rule: for each patient, only the first complete blood count obtained within the first 24 h of the index hospitalization was included, and all subsequent CBC measurements were excluded. Accordingly, each patient contributed a single observation to the dataset.

NLR, MLR, PLR, and SII values were calculated from neutrophil, lymphocyte, monocyte, and platelet counts obtained from fasting blood samples collected at 8 a.m. All complete blood-count analyses were performed according to standardized procedures in our hospital’s Central Laboratory using an automated hematology analyzer (e.g., XN-1000, Sysmex Corporation, Kobe, Japan). Blood samples were collected in tubes containing potassium ethylenediaminetetraacetic acid (K2EDTA, 367525, BD Vacutainer®, Becton Dickinson, Franklin Lakes, NJ, USA), and analyses were performed within 2 h of sample collection.

### Statistical Analysis

The statistical analyses were conducted using SPSS 22.0 (IBM Corp., Armonk, NY, USA), and since most of the data did not follow a normal distribution, the Kruskal-Wallis test was used for comparing independent groups, with Bonferroni-corrected Mann-Whitney U tests applied for pairwise comparisons. To control for Type I error across the six possible comparisons, the significance threshold was adjusted to *p* < 0.0083. The effect sizes for the Kruskal-Wallis analyses were calculated using the η^2^ formula and interpreted following Cohen’s conventions [17]. Analysis of covariance (ANCOVA) was performed, adjusting for age, sex, and length of hospital stay. The assumptions for these parametric procedures were rigorously evaluated: normality of residuals was assessed through the Kolmogorov-Smirnov test and visual inspection of Q-Q plots, homogeneity of variances was confirmed via Levene’s test, and the homogeneity of regression slopes was assessed by testing the interaction between covariates and diagnostic groups. Furthermore, multiple linear regression models were constructed to examine the effects of age, sex, and medications (lithium, antidepressants, and antipsychotics) on inflammatory indices, where multicollinearity was monitored using the Variance Inflation Factor and tolerance values to ensure model stability. In all primary analyses, a *p*-value of <0.05 was deemed statistically significant

## 3. Results

A total of 231 patients, 29 with BD-depression episode, 81 with BD-manic episode, and 121 with MDD, were found to be suitable in terms of inclusion and exclusion criteria and were included in the study. Of all patients, 57.57% were female, whereas 58.7% of the HC were female. The average age was 38.3 years in the BD-mania group, 44.3 years in the BD-depression group, 44.8 years in the MDD group, and 44.5 years in the control group. Although sex distribution and length of hospital stay did not differ significantly among groups, an omnibus test suggested a significant difference in age; however, no pairwise comparisons were significant after Bonferroni correction. Therefore, age was included as a covariate in subsequent analyses. Among the BD-mania group, 93.83% were on antipsychotics and 82.72% on mood stabilizers; in the BD-depression group, 75.86% on antipsychotics and 89.66% were on mood stabilizers; In the MDD group, 95.87% were on antidepressants. Table 1 details the age, sex, length of hospital stay, and medication use for each group. Overall, demographic characteristics and core clinical features appeared broadly comparable across the groups.

**Table 1.** **Comparison of groups in terms of age and gender**.

		BD-mania (n: 81)	BD-depression (n: 29)	MDD (n: 121)	HC (n: 121)	*p* value
Gender	Male	36 (44.4%)	12 (41.4%)	50 (41.3%)	50 (41.3%)	0.969
	Female	45 (55.6%)	17 (58.6%)	71 (58.7%)	71 (58.7%)	
Age		38.3 ± 14.0	44.3 ± 13.0	44.8 ± 15.0	44.5 ± 14.6	0.007^a^
Length of hospital stay		24.4 ± 17.5	21.8 ± 17.7	21.7 ± 16.4		0.361
Medications used	Antidepressant	0	14 (48.28%)	116 (95.87%)		
	Antipsychotic	76 (93.83%)	22 (75.86%)	46 (38.02%)		
	Mood stabilizer	67 (82.72%)	26 (89.66%)	5 (4.13%)		

^a^: For pairwise comparisons where significance was detected, the Bonferroni corrected Mann-Whitney U test was used, with the significance threshold set at *p* < 0.0083. BD, bipolar disorder; MDD, major depressive disorder; HC, healthy control.

Median neutrophil counts were numerically higher in all patient groups than in the HC group; however, only the BD-depression vs HC comparison remained significant after Bonferroni correction, while other pairwise comparisons were not significant. Lymphocyte counts were higher in the HC group than in all patient groups; however, post hoc analyses with Bonferroni correction indicated that only the MDD vs HC comparison was statistically significant, while other pairwise comparisons were not significant. Platelet counts did not differ significantly between groups in the Kruskal–Wallis test (χ^2^ = 7.656, *p* = 0.054). NLR and MLR differed significantly across groups; post hoc Bonferroni-corrected analyses (Table 2, footnote c) indicated that all patient groups had significantly higher values than the HC group. PLR showed significant group differences; post-hoc analyses indicated that only the MDD group had higher PLR values than both BD-mania and HC (χ^2^ = 39.534, *p* < 0.001; MDD > BD-mania and MDD > HC). SII values differed significantly between groups. Post-hoc tests showed higher SII in the MDD and BD-depression groups than in the BD-mania and HC groups, whereas BD-mania did not differ significantly from HC. Data regarding the group blood parameters and NLR, PLR, MLR, and SII values are presented in Table 2. NLR and MLR values were significantly higher in all patient groups than in the HC group. PLR was elevated only in the MDD group, whereas SII was increased in the BD-depression and MDD groups but did not differ between BD-mania and HC (Table 2).

**Table 2.** **Comparison of groups in terms of neutrophil, platelet, lymphocyte counts, NLR, and PLR values**.

	BD-Depression (n: 29)	BD Mania (n: 81)	MDD (n: 121)	HC (n: 121)	χ ^2^	η ^2^	*p*
	Median (IQR)	Median (IQR)	Median (IQR)	Median (IQR)			
Neutrophil	5.25 (3.94–6.40)	5.35 (3.54–6.28)	4.47 (3.28–5.77)	4.20 (3.40–4.90)	18.726	0.045	0.001^a^
Platelet	259.00 (241.00–301.00)	238.00 (214.00–277.00)	261.00 (223.00–314.00)	260.00 (220.00–315.00)	7.656	0.013	0.054
Lymphocyte	2.23 (1.93–2.80)	2.27 (1.79–2.94)	2.01 (1.56–2.38)	2.50 (2.10–3.15)	37.425	0.099	0.001^b^
NLR	2.29 (1.64–3.33)	2.34 (1.53–3.19)	2.26 (1.72–3.02)	1.50 (1.26–2.09)	43.633	0.117	0.001^c^
PLR	113.15 (97.75–169.43)	110.04 (86.17–131.49)	135.75 (105.88–174.26)	105.00 (79.49–125.71)	39.534	0.105	0.001^d^
SII	567.48 (432.40–1039.46)	542.40 (328.83–806.59)	619.88 (397.00–856.28)	399.53 (308.36–544.00)	30.103	0.078	0.001^e^
MLR	0.28 (0.22–0.34)	0.27 (0.21–0.33)	0.29 (0.23–0.35)	0.20 (0.15–0.25)	36.195	0.095	0.001^c^

NLR, neutrophil-to-lymphocyte ratio; PLR, platelet-to-lymphocyte ratio; SII, systemic immune-inflammation index; MLR, monocyte-to-lymphocyte ratio; IQR, inter quartile range. The data represent the median ranks for each group. The groups were compared using the Kruskal-Wallis test. For pairwise comparisons where significance was detected, the Bonferroni corrected Mann–Whitney U test was used, with the significance threshold set at *p* < 0.0083 (alpha = 0.05/6). ^a^: BD Depression > HC, ^b^: HC > MDD, ^c^: BD Depression, BD Mania, MDD > HC, ^d^: MDD > HC, BD Mania, ^e^: Major Depressive Disorder = BD Depression > BD Mania, HC.

The results of ANCOVA, adjusted for age, sex, and length of hospital stay, are presented in Table 3. ANCOVA revealed that the main effect of diagnostic group remained significant for all inflammatory markers after adjusting for covariates. Age showed significant associations with NLR and SII, sex with PLR, and length of hospital stay with NLR and SII. It is noteworthy that the persistence of significant group effects after covariate adjustment indicated that between-group differences were not solely driven by demographic or clinical confounders.

**Table 3.** **Results of covariate and main effect analysis for dependent variables**.

Dependent variable	Source	F value	*p* value	Partial eta squared (η^2^)
NLR	Age	4.635	0.032	0.013
	Gender	1.998	0.158	0.006
	Length of hospital stay	6.330	0.012	0.018
	Main effect (df = 3) Group	2.682	0.047	0.023
PLR	Age	0.858	0.355	0.002
	Gender	5.208	0.023	0.015
	Length of hospital stay	1.704	0.193	0.005
	Main effect (df = 3) Group	4.355	0.001	0.083
SII	Age	4.454	0.036	0.013
	Gender	0.173	0.678	0.001
	Length of hospital stay	6.013	0.015	0.017
	Main effect (df = 3) Group	3.059	0.028	0.026
MLR	Age	1.183	0.278	0.003
	Gender	0.104	0.752	0.001
	Length of hospital stay	0.602	0.439	0.002
	Main effect (df = 3) Group	13.801	0.001	0.106

According to the results of regression models examining the predictive effects of age, sex, lithium treatment, and antidepressant and antipsychotic use on NLR, PLR, and SII values, age had a significant positive predictive effect on all three variables. Multicollinearity was not an issue in the regression models, as all Variance Inflation Factor (VIF) values were well below the threshold of 5, and tolerance values were above 0.2, confirming the stability and reliability of the regression coefficients. Male sex was negatively associated with PLR (β = –0.134, *p* = 0.039), whereas antidepressant use was negatively associated with both PLR (β = –0.247, *p* = 0.002) and MLR (β = –0.165, *p* = 0.041) (Table 4).

**Table 4.** **Regression models examining the effects of drugs on NLR, PLR, MLR, and SII values**.

	NLR			PLR			MLR			SII		
	β	*t*	*p*	β	*t*	*p*	β	*t*	*p*	β	*t*	*p*
Constant		3.245	<0.001		8.130	<0.001					3.386	<0.001
Lithium use (Yes vs No)	–0.030	–0.414	0.679	–0.103	–1.477	0.141	–0.035	–0.710	0.478	–0.057	–0.795	0.428
Antipsychotic use (Yes vs No)	–0.096	–1.280	0.202	0.011	0.012	0.873	–0.091	–0.980	0.328	–0.094	–1.257	0.210
Antidepressant use (Yes vs No)	–0.106	–1.306	0.193	–0.247	–3.130	0.002	–0.165	–2.051	0.041	–0.125	–1.554	0.120
Age	0.169	2.522	0.012	0.134	2.053	0.041	0.155	2.140	0.033	0.189	2.826	0.005
Gender (Male vs Female)	0.060	0.909	0.364	–0.134	–2.080	0.039	0.042	0.615	0.539	–0.035	–0.529	0.597

## 4. Discussion

When comparing BD-depression, BD-mania, MDD, and HC groups, sex distribution and length of hospital stay were comparable across groups, whereas age differed significantly at the omnibus level; therefore, age was included as a covariate in all subsequent analyses. We found that NLR and MLR values in all patient groups were higher than those in the HC group. PLR values differed significantly across groups (Kruskal–Wallis χ^2^ = 39.534, *p* < 0.001); Bonferroni-corrected post-hoc analyses showed higher PLR values in the MDD group compared with both the BD-mania and HC groups (*p* < 0.0083). These patterns were broadly consistent with prior evidence of low-grade systemic inflammation in mood disorders and should be regarded as largely descriptive findings rather than evidence of a distinct pathophysiological mechanism. In a recent study comparing inflammatory markers in healthy controls with those in patients with schizophrenia and BD during the relapse period, NLR, PLR, SII values, and neutrophil counts were found to be higher in patients with manic episodes than in the HC group, whereas no significant difference was observed in MLR [18]. In a letter published by Korkmaz [19], inflammatory markers in patients with schizophrenia and bipolar disorder during the attack period were reported not to show any significant difference from the control group after controlling for age, sex, and active smoking. In a study by Özdin and Usta [20], neutrophil and platelet counts, along with NLR, PLR, and MLR values, were higher in patients experiencing manic episodes than in the control group; however, only MLR values remained significantly higher in the remission phase. The fact that MLR values were higher than those of controls in both euthymic and manic periods suggested that MLR may be a trait marker for BD [20].

A study examining thiol-disulfide homeostasis, ischemia-modified albumin (IMA), and CBC-derived inflammatory markers from acute mania to early remission in BD found that even when patients with acute mania achieved early remission, the thiol-disulfide parameters, IMA, and the systemic inflammatory response index (SIRI) values remained significantly different from those of healthy controls. However, NLR, SII, and CRP values became similar to those of healthy controls in early remission. These results suggested that thiol-disulfide parameters, IMA, and SIRI values may be “trait” biomarkers of inflammation in BD, whereas NLR, SII, PAR values, and CRP level may be “state” biomarkers of inflammation in BD patients in the manic phase [21]. Our findings demonstrated distinct patterns among inflammatory markers. While NLR levels were elevated in all patient groups, including those experiencing mania, SII values were increased only in the MDD and BD-depression groups, with no significant difference between the BD-mania and HC groups. This pattern suggested that NLR reflects general acute stress responses, whereas SII may relate more to depressive features. However, interpretations involving the BD-depression subgroup should be made cautiously due to the limited sample size in the present study, and the subsequent increased risk of a Type II error. It is important to note that the presence of similarly elevated NLR values across all patient groups and the episode-dependent pattern of SII should not be interpreted as evidence of a shared biological etiology. Instead, these findings likely reflect nonspecific physiological stress responses. Given the retrospective design and unmeasured confounders, including BMI, smoking status, metabolic comorbidities, and illness severity, any causal inferences regarding the relationship between inflammatory markers and mood states would be premature. Thus, although our results suggested an association between mood episodes and alterations in readily accessible hematological markers, they did not establish these parameters as mechanistic or etiological pathways. Further longitudinal studies controlling for clinical and lifestyle variables are needed to clarify the stability, specificity, and diagnostic relevance of these inflammatory markers.

Recent findings in the field have indicated that CBC-derived inflammatory markers do not exhibit a consistent, unidirectional pattern in bipolar disorder. In particular, the variation in MLR across episode types presents a heterogeneous picture in the literature [22, 23]. Therefore, the similar increase in MLR across all patient groups in our study suggested that MLR may reflect a nonspecific inflammatory response accompanying acute episodes of mood disorders rather than an episode-specific marker. This result was consistent with recent literature that reported no significant and consistent differences between episodes.

In our study, PLR value was significantly higher than the HC group only in the MDD group; a negative and significant association was found between antidepressant use and PLR and MLR values. Previous studies have reported associations between antidepressant use and lower levels of certain peripheral inflammatory markers; however, these findings are generally interpreted in the context of nonspecific systemic stress and illness severity rather than direct etiological mechanisms [24]. Although antidepressant use is generally associated with lower PLR and MLR levels, the MDD group in our study exhibited significantly higher PLR values than those in both the HC and BD-mania groups. This pattern may reflect ongoing nonspecific systemic stress or inflammatory activation commonly observed in acutely hospitalized patients, rather than a treatment-specific biological effect. Given that PLR is influenced by multiple physiological and psychosocial factors, including stress, sleep disruption, metabolic status, and chronic illness, the elevated PLR in the MDD group is more plausibly explained by nonspecific systemic processes rather than by disorder-specific inflammatory mechanisms. It is important to note that due to the cross-sectional design and the absence of standardized symptom-severity data, stronger conclusions about the relationship between PLR, antidepressant use, and underlying pathophysiology cannot be drawn.

In our study, PLR values were significantly higher only in the MDD group than in the HC and BD-mania groups, despite the negative association between antidepressant use and PLR and MLR levels. PLR findings in the recent literature were highly inconsistent, likely reflecting heterogeneity in diagnostic profiles, comorbidities, disorder characteristics, and the anti-inflammatory effects of psychotropic treatment [25, 26]. Moreover, recent studies have highlighted emerging inflammatory markers such as the neutrophil to high-density lipoprotein (HDL) ratio (NHR), lymphocyte to HDL ratio (LHR), and platelet to HDL ratio (PHR), suggesting that platelet-related indices may be influenced by various physiological and metabolic factors beyond diagnostic categories [27]. Given the retrospective, cross-sectional inpatient design and the high rate of antidepressant use in the MDD group, medication-related findings should be interpreted as exploratory. These associations are potentially confounded by indication and illness severity, and therefore do not permit causal inference.

Psychotropic drugs, including lithium and antipsychotics, influence hematologic inflammatory markers [28]. Dawidowski *et al*. [29] compared NLR values of schizophrenia patients before and after hospitalization and showed that patients who had not received antipsychotic treatment before hospitalization had significantly lower NLR values after treatment. Our study sample consisted of inpatients with mood disorders, and all participants in the patient group were using psychotropics. Only antidepressant use was found to have a negative association on MLR and PLR values, and no significant effect was found on other drugs. NLR and MLR values were markedly higher in all three patient groups than in the HC group, with no significant differences between the patient groups. In this context, our findings were consistent with the notion that acute mood episodes are accompanied by increases in inflammatory markers, despite varying diagnoses and medication profiles. This pattern may reflect state-related physiological changes during the acute phase of the disorder rather than a specific causal mechanism.

A strength of our study is the examination of mood disorders in three groups: BD-manic episodes, BD-depression episodes, and MDD. There are very few studies addressing the relationship between mood disorders and inflammatory parameters across these three groups, and we believe our study makes a significant contribution to the literature in this regard.

A significant methodological limitation of this study is that data on several key clinical variables known to have potential confounding effects on inflammatory parameters (specifically, BMI, smoking, sedentary lifestyle, and metabolic comorbidities) were not collected. These variables could not be controlled.

A second significant limitation of the study is the wide variety of medications and drug combinations used in the patient groups, and the small number of patients, particularly in the BD-depression episode group. We could not perform stratified analyses by medication type due to insufficient subgroup sizes. Medication use remained an important uncontrolled confounder. The small number of patients in the BD-depression episode group compared to the other groups significantly reduced the statistical power of the study, particularly for comparisons involving this group. Therefore, the lack of a significant difference in PLR values between the bipolar depressive group and the other groups should be interpreted cautiously, not as evidence of true no difference, but as a possible result of sample insufficiency.

Although the control group was matched to the MDD group in terms of age and sex, no such matching was performed for the BD-mania and BD-depression groups. As shown in Table 1, the BD-mania group was notably younger than the other patient groups and the healthy controls, which introduced a potential source of bias. Although ANCOVA was used to control for age and sex statistically, age showed significant associations with several inflammatory markers; therefore, residual age-related confounding could not be entirely excluded. Although ANCOVA is considered relatively robust to moderate deviations from normality, not all model assumptions (e.g., normality of residuals and homogeneity of regression slopes) were formally tested, which should be taken into account when interpreting adjusted results.

The lack of correlation analysis between inflammatory markers and standardized clinical scales measuring disease severity or treatment response (e.g., Beck Depression Inventory, Clinical Global Impression) should be considered a limitation. Our retrospective design prevented the collection of such standardized severity data from medical records, which is a fundamental limitation for interpreting our findings. Performing these analyses would have helped us understand whether these parameters are not only markers of etiology but also potential biomarkers that reflected disease severity or predicted treatment response. Additionally, we did not determine white blood cell subtypes, such as T helper 1 (Th1), T helper 2 (Th2), and natural killer (NK) lymphocytes, which limited the interpretation of the results.

Another important limitation of the study was our reliance on nonspecific inflammatory markers such as NLR and PLR, which were insufficient to support causal claims. Although these markers were convenient and cost-effective, they did not provide in-depth information on underlying mechanisms such as cytokine activity or immune cell subtypes. Furthermore, this cross-sectional study design did not allow us to distinguish whether the observed inflammation was a persistent feature of the disorder or merely a transient feature of the severe mood episode. Therefore, although our findings suggested an association, they were insufficient to conclude that inflammation plays a central or causal role in mood disorders. Additionally, the use of parametric ANCOVA despite non-normal distributions represented a methodological limitation, although the test is considered robust under such conditions.

From a broader perspective, our findings offered an integrative view of inflammation across mood disorders. By simultaneously evaluating BD-manic episode, BD-depression episode, and MDD within a unified analytical framework, this study showed that NLR and MLR elevations are comparable across all three acute diagnostic groups, whereas between-patient differences remained modest. Few studies, to date, have systematically examined these three acute mood states within a single design. Rather than supporting disorder-specific inflammatory signatures, the present results aligned with a transdiagnostic interpretation, suggesting a shared, nonspecific inflammatory upregulation associated with acute affective episodes. The present findings should not be interpreted as evidence for a shared inflammatory etiology across mood disorders, but rather as reflecting transdiagnostic state-related inflammatory changes associated with acute psychiatric hospitalization. On this basis, a plausible testable hypothesis for future work is that CBC-derived indices such as NLR, MLR, PLR, and SII may function as transdiagnostic state markers associated with episode severity or treatment response, rather than as disorder-specific etiological biomarkers.

Future research should adopt prospective, longitudinal designs that include drug-naïve or newly diagnosed patients to better differentiate episode-related inflammatory changes from stable individual traits. Rigorous age- and sex-matching across all diagnostic groups will be essential to prevent demographic confounding. Moreover, expanding data collection to encompass lifestyle and clinical covariates such as BMI, smoking, metabolic parameters, sleep patterns, and standardized measures of symptom severity would allow for more refined multivariate modeling. Systematic inclusion of emerging hematologic markers (e.g., NHR, LHR, PHR) and cytokine panels may also clarify whether CBC-derived indices reflect transdiagnostic state changes, treatment response signals, or distinct pathophysiological pathways. Together, such methodological improvements will enable clearer interpretation of inflammatory dynamics in mood disorders and support the development of more targeted biomarker-driven models.

## 5. Conclusions

This study provided one of the few integrated comparisons of inflammatory markers across three acute mood states—BD-mania, BD-depression, and MDD—within a single analytical framework. Across all diagnostic groups, NLR and MLR values were consistently higher than in healthy controls, supporting the presence of a shared, nonspecific inflammatory activation during acute affective episodes rather than disorder-specific immunological signatures. PLR elevations uniquely observed in the MDD group, together with the negative associations between antidepressant use and PLR/SII, further highlight the context-dependent and nonspecific nature of hematologic inflammatory markers in acutely ill psychiatric inpatients.

In this context, CBC-derived inflammatory indices are better conceptualized as transdiagnostic state markers observed during acute inpatient episodes rather than indicators of a shared inflammatory etiology. Although the cross-sectional design limited causal interpretation, the results underscored the potential utility of inflammatory markers in characterizing acute mood states. Future research employing longitudinal designs, drug-naïve or newly diagnosed samples, and more comprehensive clinical and lifestyle covariates will be essential to determine whether these markers hold value for predicting episode severity, treatment response, or distinct mood-disorder trajectories.

## Availability of Data and Materials

The datasets used and analyzed during the current study are available from the corresponding author upon reasonable request.
